# Increased Weight Gain and Insulin Resistance in HF-Fed PLTP Deficient Mice Is Related to Altered Inflammatory Response and Plasma Transport of Gut-Derived LPS

**DOI:** 10.3390/ijms232113226

**Published:** 2022-10-30

**Authors:** Lorène J. Lebrun, Gaëtan Pallot, Maxime Nguyen, Annabelle Tavernier, Alois Dusuel, Thomas Pilot, Valérie Deckert, Isabelle Dugail, Naig Le Guern, Jean-Paul Pais De Barros, Anissa Benkhaled, Hélène Choubley, Laurent Lagrost, David Masson, Thomas Gautier, Jacques Grober

**Affiliations:** 1 INSERM, LNC UMR1231, Université Bourgogne Franche-Comté, 21000 Dijon, France; 2 FCS Bourgogne-Franche Comté, LipSTIC LabEx, 21000 Dijon, France; 3 Institut Agro Dijon, 1 Esplanade Erasme, 21000 Dijon, France; 4 Department of Anesthesiology and Intensive Care, Dijon University Hospital, 21000 Dijon, France; 5 Faculté de Médecine Pitié-Salpêtrière, UMR1269, 75000 Paris, France; 6 Lipidomic Analytic Plate-Forme, UBFC, Bâtiment B3, 21000 Dijon, France; 7 Laboratory of Clinical Chemistry, François Mitterrand University Hospital, 21000 Dijon, France

**Keywords:** PLTP (phospholipid transfer protein), LPS (lipopolysaccharides), lipid metabolism, obesity, inflammation, dietary fat

## Abstract

Bacterial lipopolysaccharides (LPS, endotoxins) are found in high amounts in the gut lumen. LPS can cross the gut barrier and pass into the blood (endotoxemia), leading to low-grade inflammation, a common scheme in metabolic diseases. Phospholipid transfer protein (PLTP) can transfer circulating LPS to plasma lipoproteins, thereby promoting its detoxification. However, the impact of PLTP on the metabolic fate and biological effects of gut-derived LPS is unknown. This study aimed to investigate the influence of PLTP on low-grade inflammation, obesity and insulin resistance in relationship with LPS intestinal translocation and metabolic endotoxemia. Wild-type (WT) mice were compared with *Pltp*-deficient mice (*Pltp*-KO) after a 4-month high-fat (HF) diet or oral administration of labeled LPS. On a HF diet, *Pltp*-KO mice showed increased weight gain, adiposity, insulin resistance, lipid abnormalities and inflammation, together with a higher exposure to endotoxemia compared to WT mice. After oral administration of LPS, PLTP deficiency led to increased intestinal translocation and decreased association of LPS to lipoproteins, together with an altered catabolism of triglyceride-rich lipoproteins (TRL). Our results show that PLTP, by modulating the intestinal translocation of LPS and plasma processing of TRL-bound LPS, has a major impact on low-grade inflammation and the onset of diet-induced metabolic disorders.

## 1. Introduction

Obesity is a pathology whose incidence is constantly increasing worldwide. It is one of the largest world’s health problems [1] and is a major risk for serious noncommunicable disorders, including insulin resistance, type 2 diabetes, atherosclerosis and some cancers. It is now well-established that high-fat (HF) diets contribute to the development of obesity [2]. Moreover, an excess of dietary fats not only induces weight gain, but it also promotes the associated inflammatory background. Firstly, high amounts of dietary fat increase systemic exposure to potentially proinflammatory free fatty acids and their derivatives [3]. Secondly, their intestinal absorption was recently found, in both mice and humans, to facilitate the passage of bacteria-derived proinflammatory compounds such as lipopolysaccharides (LPS, endotoxins) from the gut lumen to plasma [3,4]. Finally, HF diets are also known to disturb gut barrier organization and permeability and therefore to allow the entrance of luminal compounds such as the above-mentioned endotoxins. Increased endotoxemia, which results from this trans- and paracellular intestinal translocation, triggers a slight increase in plasmatic levels of proinflammatory cytokines such as interleukin-6 (Il-6) and tumor necrosis factor-α (TNF-α) [5] and therefore participates in the onset of a low-grade metabolic inflammation. When chronic, this low-grade inflammation alters the function of several organs such as adipose tissue, muscle, liver and intestine [6]. Previous studies demonstrated that the experimental induction of metabolic endotoxemia by continuous infusion of low doses of LPS is able to mimic the metabolic consequences of a HF diet. For instance, it promotes the proliferation of adipocyte precursors, the accumulation of fat mass and the subsequent development of insulin resistance [7]. In addition, the deleterious effects of HF diets on intestinal permeability and on the development of metabolic endotoxemia have also been investigated. Indeed, it has been suggested that changes in the gut microbiota control metabolic endotoxemia, inflammation and associated disorders by a mechanism that could increase the intestinal permeability [8,9,10]. Furthermore, antibiotic treatment in mice can deeply reduce these negative effects and leads to a decreased fat mass, a lower macrophage infiltration in adipose tissue and an improved glucose tolerance but also a reduction in the LPS plasma levels [11].

In the vascular compartment, plasma lipoproteins play a major protective role during inflammation and sepsis by inactivating and eliminating LPS [12]. In the circulation, low-density lipoprotein (LDL) and high-density lipoprotein (HDL)-bound LPS can be taken up by the liver through the interaction of these lipoproteins [13] with their hepatic receptors, i.e., LDL-receptor (LDLR) and scavenger receptor B type I (SR-B1), respectively [14,15]. In addition to LDL and HDL, LPS can also be transported by chylomicrons (CM) [16]. Indeed, CM have a high affinity for LPS and thus not only transport postprandial fat but also significant amounts of concomitantly absorbed LPS. Interestingly, sequestration of absorbed LPS on CM reduces LPS toxicity and enhances its hepatic clearance [17].

In addition to lipoproteins, plasma proteins such as phospholipid transfer protein (PLTP) contribute to the control of endotoxemia [18,19,20]. PLTP was first described for its role in phospholipid transfer between lipoprotein classes [18]. In previous works, we demonstrated that PLTP plays also an important role in the reverse lipopolysaccharide transport pathway (RLT pathway) by promoting the association of LPS molecules to circulating HDL in vivo [21]. Indeed, PLTP is able to transfer and exchange LPS between circulating lipoproteins [22], thereby influencing LPS detoxification by the liver. Despite its potential beneficial impact on LPS clearance, the role of PLTP in the onset of obesity and inflammation remains controversial. Indeed, in *Pltp*-deficient mice (*Pltp*-KO), the decreased binding of LPS to HDL and, thus, detoxification from the body was associated with an increase in insulin secretion and a better oral glucose tolerance response [23]. Moreover, some studies have demonstrated an increased PLTP activity associated with obesity and metabolic syndrome in humans, whereas others have suggested that PLTP deficiency could lead to an improvement in tissue and whole-body insulin sensitivity in mice [24,25]. In addition, a recent study has shown a beneficial effect of PLTP production by brown adipose tissue (BAT) on carbohydrate and lipid homeostasis [26].

Most of the protective roles of lipoproteins and PLTP on inflammation were observed in models of acute increase in endotoxemia induced by intravenous or intraperitoneal injections of LPS. However, the impact of PLTP on inflammation induced by gut-derived LPS in a context of a HF diet and obesity is still unclear. The aim of the present study was to investigate this relationship in mice expressing or not PLTP and submitted to low-fat (LF) or HF diets. We show here that PLTP deficiency worsens HF-diet induced obesity and insulin resistance in mice through mechanisms involving alterations of plasma triglyceride (TG) clearance, the inflammatory response and lipoprotein-mediated transport of gut-derived LPS.

## 2. Results

### 2.1. Increased Fat Mass in Pltp-KO Mice under HF Diet

**Figure 1:**
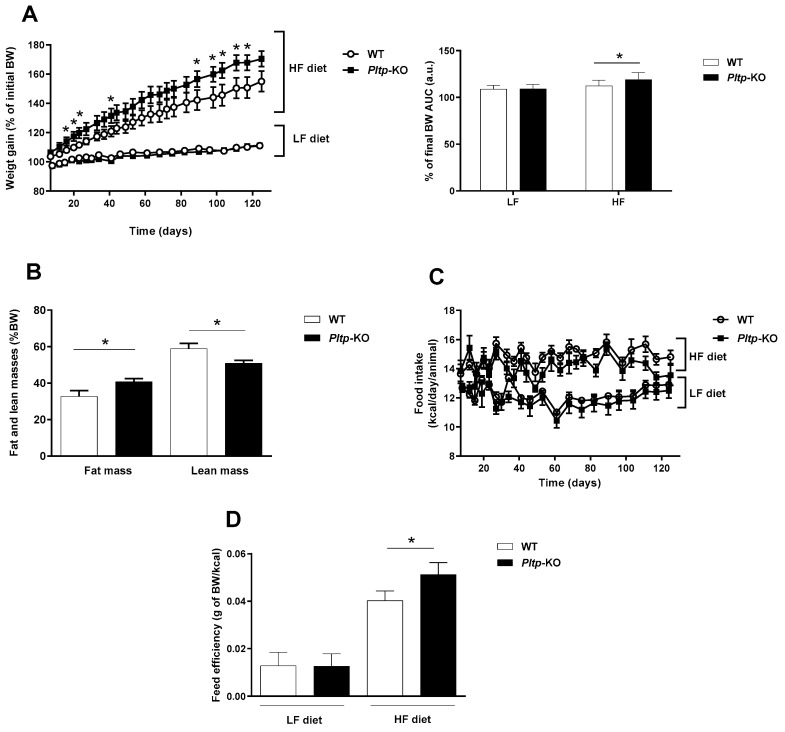
*Pltp*-KO mice have a higher fat mass under a HF diet. (**A**) Weight gain and area under the curves (AUC; a.u.: arbitrary units) of the final body weight (BW) in WT and *Pltp*-KO mice under LF and HF (*n* = 10–12). (**B**) Body composition in mice fed with a HF diet for 4 months (*n* = 10–12). (**C**) Food intake in WT and *Pltp*-KO mice under LF and HF diets (*n* = 10–12). (**D**) Feed efficiency in WT and *Pltp*-KO mice under LF and HF diets for 4 months (*n* = 10–12). Statistical analyses were performed using the Student’s *t*-test, * *p* < 0.05. All results are expressed as the mean ± SEM.

Weight gain, food intake, energy expenditure (EE) and fat mass were assessed in mice expressing active PLTP (WT) or not (*Pltp*-KO) that were fed for 4 months with a LF or HF diet (Figure 1 and Supplemental Table S1). No significant difference in body weight was observed between WT and *Pltp*-KO mice fed with LF. Under HF conditions, both WT and *Pltp*-KO mice displayed a higher weight gain than their LF counterparts. Weight gain in *Pltp*-KO mice was significantly higher than in WT mice as early as two weeks after the onset of the diet, leading to 21.7% increase in weight gain in *Pltp*-KO mice (+60.13 ± 3.14 vs. +49.44 ± 3.90% in WT mice; *p* < 0.05) after 4 months (Figure 1A, left). The area under the curves (AUC) confirmed a higher weight gain in *Pltp*-KO mice fed with HF diet (112.5 ± 5.9 vs. 119.3 ± 7.5 a.u. in WT and *Pltp*-KO mice, respectively; *p* < 0.05) (Figure 1A, right). A higher proportion of fat mass (40.7 ± 1.7 vs. 32.7 ± 3.2% of BW in WT mice; *p* < 0.05) and a lower lean mass (50.8 ± 1.68 vs. 58.9 ± 2.86% of BW in WT mice; *p* < 0.05) were observed in *Pltp*-KO mice compared with WT mice (Figure 1B). Additional analyses on tissue biopsies showed increased hepatic neutral lipid content and increased adipocyte size in visceral fat tissue of *Pltp*-KO mice (Supplemental Figure S1). Moreover, analysis of the food intake over the duration of the diet showed no significant difference between *Pltp*-KO mice and WT mice (Figure 1C). Similar fecal lipids content and EE were observed in both genotypes (Supplemental Table S1). As a consequence, a better feed efficiency in *Pltp*-KO mice (0.05 ± 0.02 g of body weight/kcal) compared to WT mice (0.04 ± 0.02 g of body weight/kcal) under a HF diet (*p* < 0.05) was observed (Figure 1D). 

### 2.2. Altered Carbohydrate Homeostasis in Pltp-KO Mice under HF Diet

**Figure 2:**
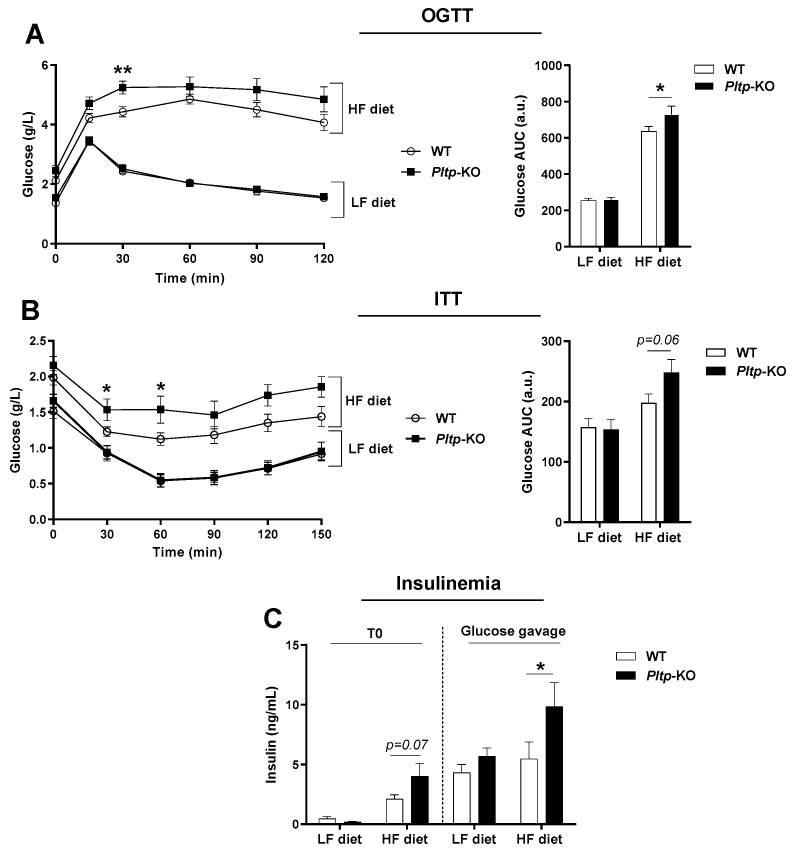
PLTP deficiency is associated with altered carbohydrate homeostasis. Evaluation of carbohydrate metabolism in WT and *Pltp*-KO mice after 4 months of a LF or HF diet. (**A**) Oral glucose tolerance test (OGTT, 1.5 g/kg), blood glucose monitoring (g/L; 120 min) and areas under the curves (AUC; a.u.: arbitrary units). (**B**) Insulin tolerance test (ITT, 0.5 u/kg), blood glucose monitoring (g/L; 150 min) and areas under the curves (AUC; a.u.: arbitrary units). (**C**) Insulin levels (ng/mL), basal (T0) and 15 min after glucose gavage at 1.5 g/kg. Statistical analyses were performed using the Student’s *t*-test, * *p* < 0.05 and ** *p* < 0.01 (*n* = 12–16). All results are expressed as the mean ± SEM.

Carbohydrate metabolism was investigated in WT and *Pltp*-KO mice (Figure 2). Under a LF diet, the oral glucose tolerance test (OGTT) led to an identical response in both genotypes. A HF diet led to higher glucose levels compared to a LF diet in both genotypes. Glucose intolerance was more pronounced in *Pltp*-KO than in WT mice (729.8 ± 45.13 vs. 635 ± 26.15 a.u., respectively; *p* = 0.03) (Figure 2A). In a similar manner, insulin tolerance test (ITT) displayed no difference under LF, an altered insulin sensitivity for both genotypes under HF and a slightly more pronounced effect in *Pltp*-KO mice (248.2 ± 21.46 vs. 197.8 ± 14.65 a.u. in WT mice; *p* = 0.06) (Figure 2B). 

The plasma insulin levels under basal conditions and after glucose gavage were measured. When comparing LF-fed mice, no differences were observed under the basal and glucose-stimulated conditions. Under a HF diet, *Pltp*-KO mice displayed a slightly higher insulinemia under basal conditions, which turned significant after glucose gavage (9.84 ± 1.98 vs. 4.03 ± 1.07 ng/mL in WT mice; *p* = 0.03) (Figure 2C). Thus, PLTP deficiency exacerbates HF diet-mediated glucose intolerance.

### 2.3. Altered Plasma Lipid Levels in Pltp-KO Mice under HF Diet

**Figure 3:**
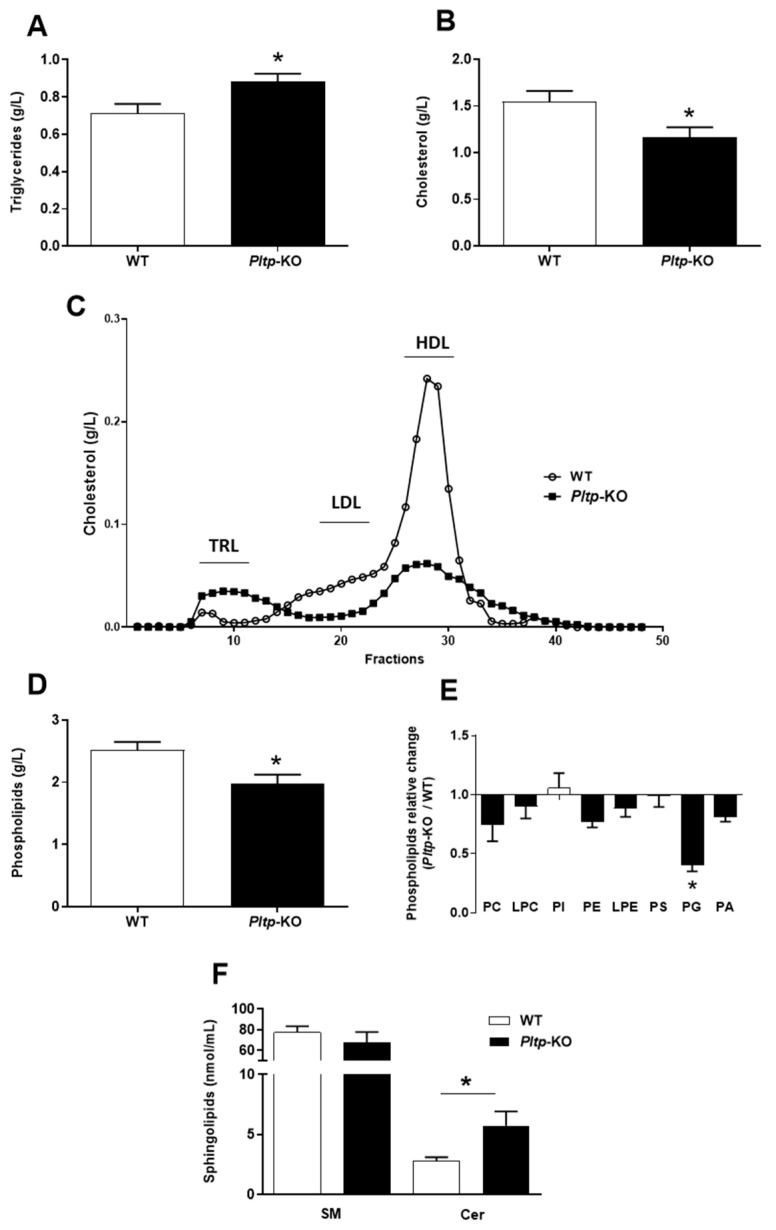
PLTP deficiency is associated with altered plasma lipid levels under a HF diet. Evaluation of plasma lipids levels in WT and *Pltp*-KO mice after 4 months of a HF diet. (**A**,**B**) Quantification of, respectively, the total plasma triglycerides and cholesterol. (**C**) Characterization of cholesterol in plasma lipoproteins (from pooled samples) by Fast protein liquid chromatography: triglyceride-rich lipoproteins (TRL), low-density lipoproteins (LDL) and high-density lipoproteins (HDL). (**D**) Quantification of total plasma phospholipid levels. (**E**) Ratios of phospholipid concentrations observed in *Pltp*-KO mice to the average of the calculated concentrations in WT mice: phosphatidylcholine (PC), lysophosphatidylcholine (LPC), phosphatidylinositol (PI), phosphatidylethanolamine (PE), lysophosphatidylethanolamine (LPE), phosphatidylserine (PS), phosphatidylglycerol (PG) and phosphatidic acid (PA). (**F**) Quantification of the total plasma sphingolipids: sphingomyelin (SM) and ceramides (Cer). Statistical analyses were performed using the matched Student’s *t*-test, * *p* < 0.05 (*n* = 10–12). All results are expressed as the mean ± SEM.

On a LF diet, the plasma cholesterol levels tended to be lower in *Pltp*-KO mice than in WT mice (0.41 ± 0.09 vs. 0.68 ± 0.13 g/L, NS), together with significant decreases in the phospholipid (0.60 ± 0.07 vs. 1.12 ± 0.07 g/L, *p* < 0.005) and sphingomyelin levels (26.9 ± 4.5 vs. 42.2 ± 3.5 nmol/mL, *p* < 0.05). *Pltp*-KO mice and WT mice showed similar levels of plasma TG (0.65 ± 0.07 and 0.71 ± 0.08 g/L, respectively, NS) and ceramide (3.3 ± 0.7 2.9 ± 0.4 nmol/mL, respectively, NS) on a LF diet. Under HF conditions, the TG levels were significantly higher in *Pltp*-KO compared to WT mice (0.88 ± 0.04 vs. 0.71 ± 0.05 g/L, respectively; *p* < 0.05) (Figure 3A), whereas the plasma cholesterol was lower (1.16 ± 0.11 vs. 1.55 ± 0.11 g/L, respectively; *p* < 0.05) (Figure 3B). A decrease in plasma cholesterol was a consequence of a lower amount in LDL and HDL fractions (Figure 3C). Plasma phospholipid profiling revealed a significant reduction in the total phospholipids in *Pltp*-KO mice (1.98 ± 0.14 vs. 2.52 ± 0.13 g/L in WT mice; *p* < 0.05) (Figure 3D), especially with a marked decrease in phosphatidylglycerol (PG) (−59.8%; *p* < 0.05) (Figure 3E). Within sphingolipids species, the ceramide levels were almost doubled in *Pltp*-KO mice (5.73 ± 1.20 vs. 2.79 ± 0.32 nmol/mL in WT mice; *p* < 0.05) (Figure 3F). These data confirm the prominent role of PLTP in plasma lipid homeostasis, which extends beyond phospholipids to ceramides.

**Figure 4:**
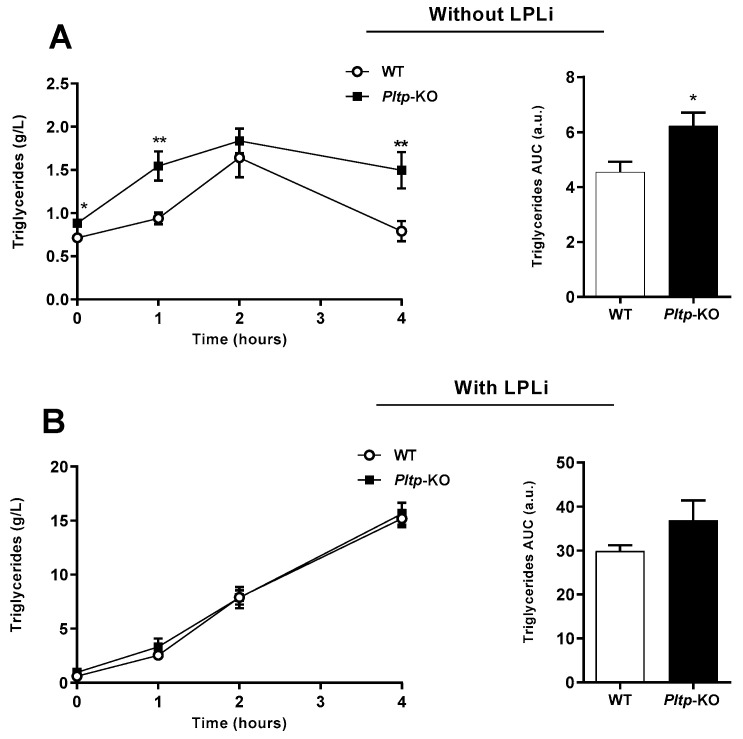
*Pltp*-KO mice have an altered triglyceride clearance under a HF diet. Quantification of plasma triglycerides (g/L) in WT and *Pltp*-KO mice fed with a HF diet for 4 months, treated or not with a lipoprotein lipase inhibitor (LPLi; poloxamer i.p. injection 407 at 1 mg/kg). (**A**) Kinetics of triglyceridemia and area under the curves (AUC; a.u.: arbitrary units) of triglyceridemia without LPLi (*n* = 11 to 12). (**B**) Kinetics of triglyceridemia and area under the curves (AUC; a.u.: arbitrary units) of triglyceridemia with LPLi (*n* = 11 to 12). Statistical analyses were performed using the paired Student’s *t*-test, * *p* < 0.05 and ** *p* < 0.01. All results are expressed as the mean ± SEM.

The oral lipid loading test led to a higher and extended TG peak in *Pltp*-KO mice compared with WT mice, with a higher TG AUC (6.23 ± 0.48 vs. 4.55 ± 0.38 a.u., respectively; *p* < 0.05) (Figure 4A). Importantly, when TG clearance was blocked with a lipoprotein lipase (LPL) inhibitor (LPLi), no differences in triglyceridemia were observed between the two genotypes (Figure 4B), indicating a key role of PLTP in TG removal from plasma.

### 2.4. Increased Inflammation and Circulating LPS and Altered Plasma Transport of LPS in Pltp-KO Mice 

**Figure 5:**
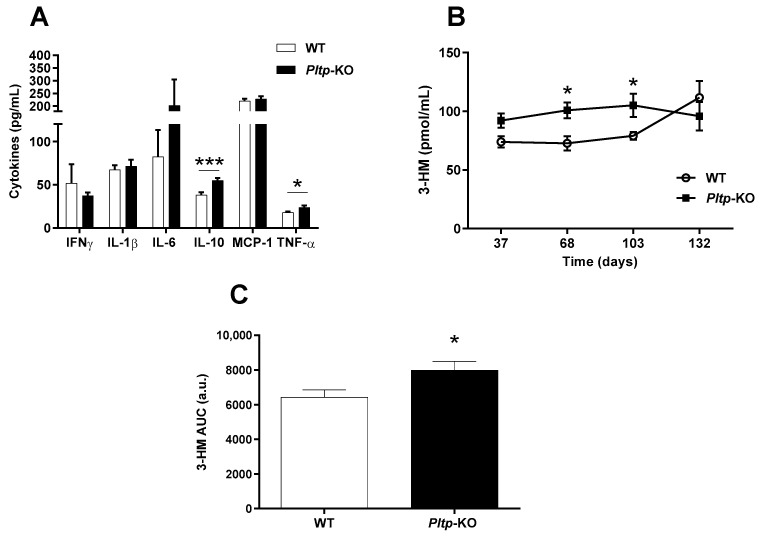
*Pltp*-KO mice have a higher inflammatory response under a HF diet. Evaluation of the inflammatory status of WT and *Pltp*-KO mice fed with a HF diet for 4 months. (**A**) Levels of cytokines (pg/mL) in mice (*n* = 12). (**B**) Quantification of circulating LPS via the measurement of 3-hydroxymyristate (3-HM; ng/mL) by HPLC/MS/MS (*n* = 19–22). (**C**) Area under the curves (AUC; a.u.: arbitrary units) of circulating LPS (*n* = 19–22). Statistical analyses were performed using a matched Student’s *t*-test, * *p* < 0.05 and *** *p* < 0.001. All results are expressed as the mean ± SEM.

The inflammatory profiles were investigated in WT and *Pltp*-KO mice under a HF diet. Plasma IL-10 and TNF-α were significantly increased in *Pltp*-KO mice when compared with WT mice (54.9 ± 2.8 vs. 37.9 ± 3.3 pg/mL for IL-10, 23.7 ± 2.34 vs. 18.1 ± 1.05 pg/mL for TNF-α; *p* < 0.001 and *p* < 0.05, respectively), demonstrating an increased response to an inflammatory stimulus in the context of a HF diet (Figure 5A). Measurements of the plasma levels of 3-hydroxymyristate (3-HM), i.e., a specific marker of LPS, showed an increased LPS concentration in *Pltp*-KO mice (Figure 5B). This was confirmed by calculation of the AUC (6429 ± 429 vs. 7996 ± 503 a.u. in WT and *Pltp*-KO mice, respectively; *p* = 0.023) (Figure 5C). 

**Figure 6:**
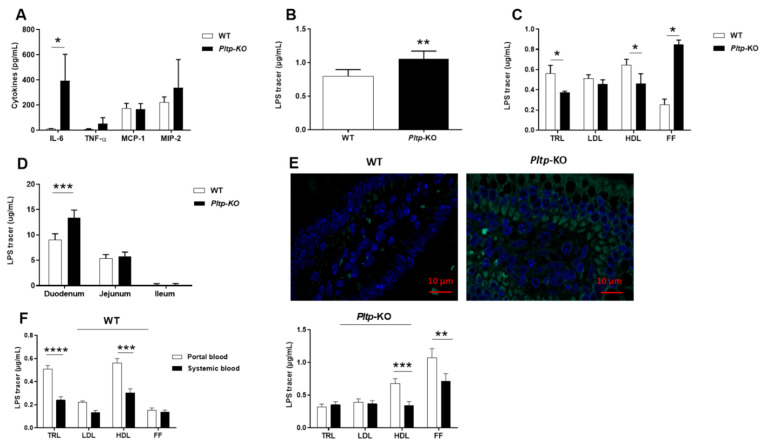
*Pltp*-KO mice have a higher inflammatory response after LPS gavage. Evaluation of the inflammatory parameters after LPS gavage (0.5 mg/kg) in WT and *Pltp*-KO mice. (**A**) Levels of cytokines (pg/mL; *n* = 7) 1 h after LPS gavage. (**B**) Plasma LPS quantification (µg/mL; *n* = 6) 1 h after LPS gavage. (**C**) Distribution of LPS (µg/mL; *n* = 6) in plasma lipoprotein fractions: triglyceride-rich lipoproteins (TRL), low-density lipoproteins (LDL), high-density lipoproteins (HDL) and free fractions (FF). (**D**) Mucosa LPS quantification (µg/mL; *n* = 6) 1 h after LPS gavage. (**E**) Immunofluorescence microscopy of WT (left) and *Pltp*-KO (right) mice small intestine (duodenum) cross-section (immersion objective x63). (**F**) Distribution of LPS (µg/mL) in portal and systemic blood 15 min after LPS gavage (*n* = 9). Statistical analyses were performed using the matched Student’s *t*-test or a two-way ANOVA analysis; * *p* < 0.05, ** *p* < 0.01, *** *p* < 0.001 and **** *p* < 0.0001. All results are expressed as the mean ± SEM.

Inflammatory profiles were also investigated in WT and *Pltp*-KO mice after an oral gavage of LPS under a LF diet. IL-6 was significantly increased in *Pltp*-KO mice (392.05 ± 80.30 pg/mL) compared to WT mice (10.01 ± 3.23 pg/mL; *p* < 0.05) (Figure 6A). Higher plasma LPS levels were observed in *Pltp*-KO vs. WT mice (1.06 ± 0.05 vs. 0.80 ± 0.04 µg/mL, respectively; *p* = 0.0017) (Figure 6B). One hour after LPS gavage, free LPS (i.e., found in the lipoprotein-free fraction, FF) was significantly higher in *Pltp*-KO mice compared to WT mice (0.85 ± 0.02 vs. 0.25 ± 0.02 µg/mL, respectively; *p* < 0.05) (Figure 6C). In addition, LPS concentration in the duodenum mucosa was 47.7% higher in *Pltp*-KO mice than in WT mice (13.37 ± 0.63 vs. 9.05 ± 0.48 µg/mL, respectively; *p* < 0.001) (Figure 6D). Microscopic observations confirmed the results obtained after fluorometric assays, with a higher fluorescent signal in the proximal sections of the small intestine in *Pltp*-KO mice (Figure 6E). Next, LPS plasma distribution was determined in portal and systemic blood at an early time point after LPS gavage (15 min). In WT mice (Figure 6F, left), the level of triglyceride-rich lipoprotein (TRL)-bound LPS was significantly decreased in systemic blood compared to portal blood (0.24 ± 0.08 vs. 0.51 ± 0.09 µg/mL in systemic and portal blood, respectively; *p* < 0.0001). The same phenomenon could be observed when considering HDL-bound LPS (0.30 ± 0.11 vs. 0.56 ± 0.11 µg/mL in systemic and portal blood, respectively; *p* = 0.0005). In *Pltp*-KO mice (Figure 6F, right), a similar decrease in HDL-bound LPS was observed from portal to systemic blood (0.67 ± 0.23 vs. 0.34 ± 0.19 µg/mL in portal and systemic blood, respectively; *p* = 0.0008). However, regarding TRL-bound LPS, no decrease was observed in *Pltp*-KO mice, while a significant drop occurred when considering free LPS (1.07 ± 0.42 vs. 0.71 ± 0.35 µg/mL in portal and systemic blood, respectively; *p* = 0.0082). Overall, these results indicate that a PLTP deficiency exacerbates the inflammatory status through an altered plasma transport of LPS. 

### 2.5. Decreased LPL Activity in Pltp-KO Mice

**Figure 7:**
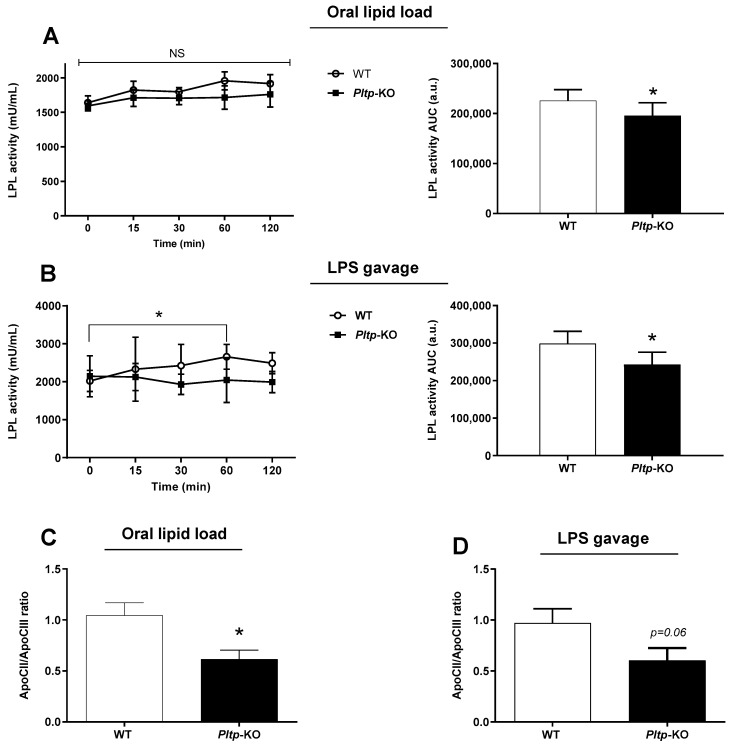
PLTP deficiency is associated with a lower LPL activity in mice. Kinetics of LPL activity and determination of the apoCII/apoCIII ratio after oral lipid loading (10 mL/kg) or LPS gavage (0.5 mg/kg) in mice. (**A**) Kinetic and AUC of LPL activity (U/mL) after corn oil gavage in mice (*n* = 8). (**B**) Kinetic and area under the curves (AUC; a.u.: arbitrary units) of LPL activity (U/mL) after LPS gavage (*n* = 8). (**C**) ApoCII/apoCIII ratio in mice after oral lipid loading (*n* = 10 to 11). (**D**) ApoCII/apoCIII in mice after LPS oral administration (*n* = 10 to 11). Statistical analyses were performed using the matched Student’s *t*-test, NS: not significant and * *p* < 0.05. All results are expressed as the mean ± SEM.

Plasma LPL activity was quantified after oral lipid or LPS loading (Figure 7). A 13% decrease in *Pltp*-KO mice compared to WT mice was observed after lipid gavage (*p* = 0.044), (Figure 7A) and a 18% decrease in *Pltp*-KO mice compared to WT mice was observed after the LPS oral load (*p* = 0.020) (Figure 7B). The plasma levels of apolipoprotein (apo) CII and apoCIII (LPL modulators) were determined in different conditions. The apoCII/apoCIII ratio was significantly decreased after an oral lipid load in *Pltp*-KO mice (0.61 ± 0.08 vs. 1.04 ± 0.12 in WT mice; *p* = 0.0122), and a similar trend was observed after the oral LPS challenge (0.60 ± 0.12 vs. 0.97 ± 0.14 in WT mice; *p* = 0.0619) (Figure 7C,D).

**Figure 8:**
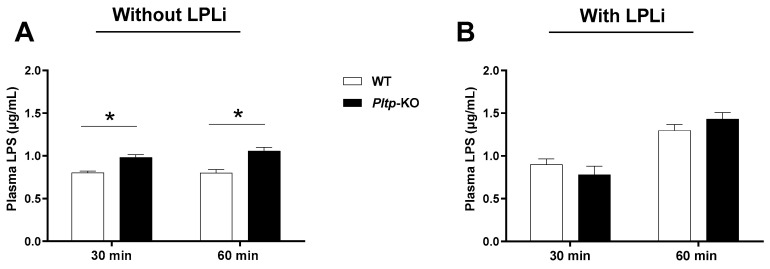
LPL inhibitor (LPLi) abrogates the modulatory effect of PLTP on the LPS plasma levels. Kinetics of the plasma LPS levels (µg/mL) in mice 30 and 60 min after LPS oral administration. (**A**) The plasma LPS levels after LPS gavage (*n* = 8). (**B**) The plasma LPS levels after LPLi (P407) i.p. injection and LPS gavage (*n* = 8). Statistical analyses were performed using the matched Student’s *t*-test, * *p* < 0.05. All results are expressed as the mean ± SEM.

Finally, while gut-derived LPS accumulated to a larger extent in *Pltp*-KO mice after LPS gavage (+22.5% and +32.9% at 30 and 60 min, respectively (Figure 8A), the administration of LPLi abrogated the increased endotoxin levels in *Pltp*-KO mice (Figure 8B) indicating that the altered LPS clearance associated with PLTP deficiency was mediated by a decrease in LPL activity.

## 3. Discussion

Here, we report the deleterious consequences of PLTP deficiency, linked to the exacerbated response to a HF diet, leading to higher adiposity, as well as worsened glucose and insulin tolerance. This was associated with marked alterations of plasma lipid levels and decreased postprandial TG clearance. Finally, *Pltp*-KO mice display a more pronounced inflammatory status under HF conditions, together with increased exposure to gut-derived LPS.

Increased weight gain in HF-fed *Pltp*-KO mice is not due to a higher food intake. The feed efficiency calculations indeed demonstrate that *Pltp*-KO mice are more efficient in transforming ingested kilocalories into grams of body weight. Since the fecal lipid contents appear to be identical between the two genotypes, this better efficiency is not due to a better energy absorption capacity. Although a lower EE in the absence of PLTP could be expected, no differences were observed for this parameter. However, the measurements of EE were performed in specific cages in which mice were individualized. In addition, data acquisition was done over a 24-h period, which does not fully reflect the long-term effects of a four-month diet. Moreover, possible differences in the gut microbiota-produced energy [27,28], which could be modified in the absence of PLTP, have been overlooked in our EE measurements. Lastly, compared to WT, *Pltp*-KO mice display a higher adipose (low metabolic activity) to lean (high metabolic activity) mass ratio. Although this suggest that EE measurements need to be adjusted to this morphological disparity, there are still controversies about the most relevant calculation method [29].

*Pltp*-KO mice have a higher amount of total fat tissues, adipocytes hypertrophy in visceral fat and higher hepatic lipid accumulation under HF conditions. The expansion of adipose tissue and liver steatosis are related to lipotoxicity, which, in turn, is detrimental for insulin sensitivity [30,31,32], resulting in worsened glucose metabolism. Thus, given that *Pltp*-KO mice become fatter on a HFD, it is not surprising that they exhibit more pronounced alterations in glucose control.

*Pltp*-KO mice on a LF diet did not show significant differences in plasma TG levels. The trend to lower plasma cholesterol, as well as decreased plasma phospholipid and sphingomyelin levels in *Pltp*-KO mice on a LF diet, have already been described elsewhere [33,34]. Interestingly, lower sphingomyelin would rather indicate protection against insulin resistance and inflammation in this context [35]. On a HF diet, however, the lipid profile of *Pltp*-KO is in line with their insulin-resistant status compared to WT mice.. Previous studies in *Pltp*-KO mice described a reduced production of TRL [36]. Since it is known that a HF diet increases the TG levels in response to a decreased insulin sensitivity in mice [4,37], our observations suggest that increased plasma TG levels in our *Pltp*-KO model might well be the consequence of altered insulin sensitivity after a HF diet. In addition, the absence of PLTP is also associated with an increase in the ceramide levels, which are known to be closely related to the pathogenesis of insulin resistance [38]. Moreover, *Pltp*-KO mice display decreased HDL cholesterol levels, which is also a factor associated with a loss of insulin sensitivity [39]. The interpretation of changes in the phospholipid profiles in *Pltp*-deficient mice is more difficult. Although all phospholipid classes tend to decrease, we noticed a marked effect of PLTP deficiency on phosphatidylglycerols (PG), which are among the lowest abundant phospholipids in the plasma. The positive association of PG levels with fat mas was reported in obese patients [40], which is not seen here in fatter *Pltp*-KO mice. Decreased PG levels in *Pltp*-KO mice are also surprising with regard to their fatty liver phenotype, as PG elevation was observed in obese patients with nonalcoholic steatohepatitis (NASH) [41]. Thus, we suggest that PLTP might have a specific preference for plasma PG remodeling independent of the obesity-related metabolic status. Interestingly, recent findings suggest that PG could have an anti-inflammatory effect by inhibiting toll-like receptor-4 involved in the development of low-grade metabolic diseases [42]. The reduced PG levels in *Pltp*-KO mice might thus contribute to their increased inflammatory status. Ceramides are also known to be closely linked to inflammation. Therefore, the high ceramides plasma levels observed in *Pltp*-KO mice are likely a determinant of their higher inflammatory state [43].

Beyond the global proinflammatory lipid profiles, we documented a PLTP-dependent effect of proinflammatory LPS. HF-fed *Pltp*-KO mice have an overall increased exposure to LPS over the duration of the diet, although the difference in endotoxemia was not significant when measured in the post-absorptive state at a single time point at the end of the study. This observation highlights the importance of the regular monitoring of plasma LPS levels to clearly assess the extent of metabolic, low-grade endotoxemia [44]. Although *Pltp*-KO mice were reported to display a lower inflammatory status under basal conditions [45], PLTP deficiency is known to be associated with an increased inflammation in response to the LPS challenge [20]. Besides previously performed i.p. or i.v. injection of LPS, we show here that either oral gavage or a HF diet increase LPS in *Pltp*-KO mice. Increased LPS levels could result from a higher translocation from the gut, which is known to be modulated by a HF diet [3,4] or altered detoxification of circulating LPS [20]. To gain more insights, we developed a strategy based on the oral administration of fluorescent LPS allowing accurate monitoring in the different body compartments [46]. Our observation of higher LPS concentrations and a fluorescent signal in the proximal gut segments of *Pltp*-KO mice suggests an accelerated translocation of endotoxins. Whether a transcellular or a paracellular way for LPS crossing of the intestinal barrier [47] is affected remains to be determined. An analysis of the plasma LPS distribution revealed a lack of association with TRL despite increased TG levels in *Pltp*-KO mice. This might suggest that the modalities of LPS translocation (through packaging together with the CM in the transcellular pathway) is altered in *Pltp*-KO mice. Further studies need to be performed to precisely determine the mechanisms involved in LPS translocation in HF-fed mice. 

In our model, circulating LPS was bound to HDL and TRL in WT mice, whereas they were mainly found in the free fraction in *Pltp*-KO mice. This is in agreement with the well-known ability of PLTP to transfer LPS onto lipoproteins, especially to HDL [48]. However, we highlight here that this phenomenon occurs very rapidly upon LPS administration and, most interestingly, that this PLTP-facilitated mechanism also applies to gut-derived LPS binding to TRL and HDL. Previous studies have already demonstrated the protective role of TRL in preventing the negative consequences of endotoxemia [49,50]. Since lipoprotein-bound LPS are considered an inactive form [51], the defective association of gut-derived LPS to lipoproteins is likely a proinflammatory contributor in *Pltp*-KO mice. Besides inactivating circulating LPS, lipoproteins also play a key role in promoting the hepatic-mediated clearance of LPS [14]. Our observations reporting significant portal to central decreases in TRL- and HDL-bound LPS in WT mice are in line with the concept. Importantly, *Pltp*-KO mice displayed an altered hepatic clearance of TRL-bound LPS. This might relate to an impaired LPS binding to TRL despite increased TG levels or to an impaired TRL clearance.

We demonstrate that LPL activity is decreased in *Pltp*-KO mice either after an oral lipid load or after an oral LPS administration. Moreover, an exacerbated response to the oral lipid load in *Pltp*-KO mice was blunted when a LPL inhibitor was administered. These results suggest that TRL catabolism is indeed impaired in HFD *Pltp*-KO mice. In order to gain more insight into the underlying causes of altered LPL activity in *Pltp*-KO mice, we studied the roles of apoCII and apoCIII, the LPL cofactors [52,53], and we showed that the apoCII/apoCIII balance is altered in *Pltp*-KO mice. Since HDL can be generated by LPL-mediated CM hydrolysis, it can be hypothesized that a decreased LPL activity could indirectly affect the association of bacterial toxins with HDL in *Pltp*-KO mice. Finally, the use of LPLi in our animals abrogated the difference in LPS clearance between *Pltp*-KO mice and WT mice, reinforcing the hypothesis that altered LPL activity is a leading cause of decreased LPS detoxification in our model.

Overall, our study demonstrates that PLTP deficiency leads to less efficient detoxification of LPS but, also, to an exacerbated inflammatory response in the context of obesity and insulin resistance. These observations are in accordance with the concept of metabolic endotoxemia playing a key role in the aggravation of metabolic abnormalities [8]. This work highlights the relationship between PLTP, TRL metabolism and low-grade inflammation, as observed in metabolic diseases. Beyond the well-established implication of LPL activity in obesity and type 2 diabetes [54], our results suggest that LPL also plays an important role in the detoxification of bacterial toxins and the attenuation of inflammation. The underlying causes of altered LPL modulation in *Pltp*-KO mice will need to be addressed in future studies.

## 4. Materials and Methods

### 4.1. Experimental Animals, Diets and Samplings

All experiments were performed using male wild-type (WT) C57BL/6J mice and male mice knocked out for PLTP (*Pltp*-KO) on a C57BL/6J background. Mice were matched by age (8–12 weeks old) and were housed in a controlled environment (light from 7 a.m. to 7 p.m., with a constant humidity and temperature) and were given a standard chow low-fat diet for 12 weeks (10% fat, D1250B, Research Diets Inc., New Brunswick, NJ, USA) or high-fat diet for 12 weeks (60% fat, D12492, Research Diets Inc., New Brunswick, USA). Every sampling procedure (except caudal sampling) was performed under inhaled anesthesia (isoflurane) titrated to maintain spontaneous breathing. Blood was collected simultaneously in the portal vein and by intracardiac puncture in EDTA-coated tubes. Plasmas were separated by centrifugation at 8000× *g* for 10 min at 4 °C and were stored at −20 °C until use.

### 4.2. Measurements of Weight Gain and Food Intake, Lean and Fat Masses, Energy Expenditure (EE) and Fecal Lipids Content

Body mass and food intake were measured every week in mice fed the HF and LF diets. EchoMRI^®^ was used to determine the body composition through nuclear magnetic resonance (NMR) spectroscopy (fat mass and lean masses). EE measurements were performed by indirect calorimetry and adjusted according to Even and Nadkarni [55]. First, animals are weighed and then placed in a specific containment tube (EchoMRI 500T; EchoMRI, Houston, TX, USA). Then, the tube is inserted into the device. The measurement sequence is initiated and lasts three minutes. Measurements were performed on a computer-controlled open-circuit CLAMS system (Oxymax Comprehensive Lab Animal Monitoring System; Columbus Instruments). In order to limit the effects of stress due to the modification of their environment, the mice were acclimatized to the test protocol (calorimetry cage, ventilation, etc.) for 48 h prior to the experiment. The measurement of the EE was carried out during 24 h, respecting the day/night cycle and with an air flow rate of 0.6 L/min. Gases evacuated from the chambers were sampled for 45 s every 20 min after a 90-s purging period. The volumes of oxygen (VO_2_) consumed and carbon dioxide (VCO_2_) produced were quantified by analyzing the sampled gases through the oxygen and carbon dioxide detector. Before starting the experiment, a calibration of the detectors was performed using a specific calibration mixture containing both oxygen, carbon dioxide and nitrogen (Air Liquide). A reference measurement with ambient air was performed every 8 measurements. The ratio of carbon dioxide production and oxygen consumption was calculated (RQ: respiratory quotient or RER: respiratory exchange ratio). At the end of the experiment, the body composition data previously measured by EchoMRI^®^ allowed the adjustment of the EE (heat production: (3.815 + 1.232 × RQ) × VO_2_) to the metabolically active mass of animals (Lean mass + 0.2 Fat mass). During the experiment, moves of the animals inside the cage were measured, as well as the consumption of water and food in real time. Total lipids in the feces were extracted by Folch’s method [56]. Briefly, this is a simple and reliable method for the extraction of fecal lipids—more particularly, phospholipids and neutral lipids such as tripalmitin, lecithin, calcium stearate, cholesterol, cholesteryl palmitate and a mixture of all of these. The fecal samples are homogenized with a mixture of chloroform, methanol, acetic acid and water. The extract is washed with water to remove the non-lipidic impurities extracted by the solvents. The washing procedure also removes the methanol and acetic acid, leaving the lipids in the chloroform layer. The recoveries of the known lipids mentioned above added to the fecal samples before extraction averaged 99%.

### 4.3. Oral Glucose Tolerance Test (OGTT) and Insulin Tolerance Test (ITT)

Mice were fasted 6 h before starting the experiments. Glucose solution (150 or 200 g/L in drinking water; G8270, SigmaAldrich, Saint Quentin Fallavier, France) or insulin solution (0.03 or 0.05u/mL in NaCl; Humalog 100 IU/mL, Lispro, Lilly) were administered at a rate of 1.5 or 2 g/kg (OGTT) and 0.3 or 0.5 u/kg (ITT), respectively. Glucose was administered orally through a cannula of gavage, and insulin was injected via IP. Blood glucose was measured over time through the tail vein and with a blood glucose monitor (OneTouchUltra, Lifescan, Boulogne Billancourt, France). Measurements were taken before gavage and 15, 30, 60, 90, 120 and 210 min after gavage for OGTT. Measurements was taken before injection and 30, 60, 90, 120 and 150 min after injection.

### 4.4. Conjugation of DOTA-Bodipy-NCS to LPS

DOTA-Bodipy-NCS was prepared using the method previously described (Bernhard et al. 2010) [57]. Briefly, LPS from *Escherichia coli* O55:B5 (Sigma Aldrich, Saint Quentin Fallavier, France) were incubated in the presence of one equivalent of DOTA-Bodipy-NCS solubilized in DMSO for 1 h at 37 °C in a hot water bath. Labeled LPS were purified on a Superdex 75 column (GE Healthcare, Marlborough, MA, USA), concentrated with Nanosep MWCO 3 kDa (Pall).

### 4.5. LPS Quantification

Concentrations of fluorescently labeled LPS in plasma and intestinal fractions were determined by the measurement of the fluorescence using a Perkin Elmer Wallac 1420 Victor2 microplate reader (absorption: 485 nm/emission: 535 nm). The total LPS concentrations were also measured by quantifying 3-OH-tetradecanoic acid (3HM) by liquid chromatography coupled with tandem mass spectrometry (LCMS2), as previously described [58]. Briefly, the samples were diluted in 8-M hydrochloric acid solution together with 3-OH-tridecanoic acid (internal standard) and hydrolyzed for 3 h at 90 °C. Free fatty acids were extracted with distilled water and a mix of hexane/ethyl acetate (3/2 *v*/*v*). The organic phase was discarded by evaporation. The dried extracts were solubilized in ethanol and injected on a SBC18 column (2.1 × 50 mm, 1.8 μm) coupled with an Infinity 1290 HPLC device (Agilent Technologies, Santa Clara, USA). An elution gradient with 5 mM ammonium formate/0.1% formic acid (eluent A) and 95% acetonitrile (eluent B) was used to separate 3OH-free fatty acids. Separated 3OH-free fatty acids were detected by tandem mass spectrometry with a QqQ 6490 triple quadruple mass spectrometer (negative mode) using a JetStream ESI source.

### 4.6. Drugs Administration in Mice

For the LPS translocation studies, mice were gavaged with Dotaga-Bodipy-LPS concentrated at 0.5 mg/kg. For TG production kinetics, mice were treated with a single dose of 0.5 g/kg Poloxamer 407 (Lutrol^®^, LPL inhibitor, Sigma Aldrich, Saint Quentin Fallavier, France) 30 min before the experiment via intraperitoneal injection to inhibit triglyceride hydrolysis. Mice were gavaged with corn oil at 10 mg/kg (Sigma Aldrich, Saint Quentin Fallavier, France).

### 4.7. Separation of Plasma Lipoproteins

TRL (i.e., CM and very low-density lipoprotein (VLDL), d < 1.006), LDL (1.006 < d < 1.063), HDL (1.063 < d < 1.21)) and the free fraction (FF) were separated using a 3-step sequential ultracentrifugation procedure in a TLA 100.1 rotor in an Optima^TM^ MAX-XP Ultracentrifuge (Beckman, Palo Alto, CA, USA). Lipoproteins were also separated by fast protein liquid chromatography (FPLC) performed on pooled plasma injected in an agarose Superose 6 column and eluted in Tris/Saline/EDTA buffer. 

### 4.8. Plasma Biochemical Analyses

Cytokines, insulin, apolipoprotein CII (apoCII) and apolipoprotein CIII (apoCIII) concentrations were determined by commercially available ELISA kits (M-CYTOMAG-70 K, Millipore; 80-INSMR-CH10, Alpco; MBS923729-96, Mybiosource; MBS944394-96, Mybiosource). The protein concentration was determined to perform normalization on samples such as the gut segments. It was measured by a commercial colorimetric assay kit (Thermo Scientific™ Pierce™ BCA assay kit). Post-heparin plasma (50 U/kg) LPL activity was measured by a commercial fluorometric kit (STA-610, Cell Biolabs, Inc., San Diego, CA, USA). Triglyceridemia and cholesterolemia were measured by commercial photometric assay kits (Triglycérides FS, DiaSys Diagnostic Systems GmbH and Cholesterol FS, DiaSys Diagnostic Systems GmbH, respectively). Phospholipid and sphingolipid profiles were determined using the method described in Anjani et al. [41].

### 4.9. Tissue Analyses

Intestinal sections (duodenum, jejunum and ileum) from mice were put in paraformaldehyde solution 4% (Cell Store Pot, 10% neutral buffer formalin, 60 mL). Intestinal sections were then cut, fixed in acetone at −20 °C for 10 min and mounted in Vectashield fluorescent mounting medium with DAPI (Vector). Slides were observed with an Axio Imager M2 epifluorescence microscope (Zeiss) with excitation at 485 nm and emission at 535 nm for Bodipy.

### 4.10. Statistics

Data were collected using Microsoft Excel for Office 365. All data were shown as the mean values ± SEM. Prism 6.0 software (GraphPad, San Diego, CL, USA) was used to perform the statistical analyses. The Shapiro–Wilk test was used to test the normality of the value distribution before performing parametric or nonparametric statistical analyses. The distributions were systematically considered as non-normal when *n* < 7. Mann–Whitney *U* test or Student’s *t*-test were used to assess the significance of differences between two groups, and a correction was applied when the groups had different variances. In the presence of more than two groups (e.g., kinetics), two-way ANOVA, followed by Tukey’s multiple comparisons was performed, if applicable. A value of *p* < 0.05 was considered statistically significant (NS, not significant; * *p* < 0.05, ** *p* < 0.01, *** *p* < 0.001 and **** *p* < 0.0001).

## Data Availability

The authors confirmed that the data supporting the findings of this study are available within the article and/or its supplementary materials.

## Supplementary Materials

The following supporting information can be downloaded at: https://www.mdpi.com/article/10.3390/ijms232113226/s1: Table S1. Energy balance of *Pltp*-KO mice is not altered under a HF diet. Figure S1. Tissue analyses (liver and adipose tissue) in WT and *Pltp*-KO mice under LF or HF.
