# Supplementary material for: Increased Weight Gain and Insulin Resistance in HF-Fed PLTP Deficient Mice Is Related to Altered Inflammatory Response and Plasma Transport of Gut-Derived LPS
Source: Int J Mol Sci. 2022 Oct 30;23(21):13226. doi: 10.3390/ijms232113226 (PMC9654699; doi:10.3390/ijms232113226)
Supplement: Supplementary file 1 [file ijms-23-13226-s001.zip › ijms-1963687-SI.pdf]

International Journal of
Molecular Sciences

MDPI

Supplementary Material

1. Table

Table S1. Energy balance of *Pltp*-KO mice is not altered under HF diet. Assessment of energy balance in WT and *Pltp*-KO mice after 4 months of HF diet: energy expenditure (EE) (kcal/day/animal) and fecal lipids content (mg/g). Statistical analyses were performed using the Student's *t* Test. All results are expressed as mean ± SEM.

	WT	*Pltp*-KO	n (per group)	*p* values
Energy expenditure (EE)	14.93±0.60	16.62±0.87	12	0.12
Fecal lipids content	0.02±0.01	0.02±0.01	4	0.60

2. Figure

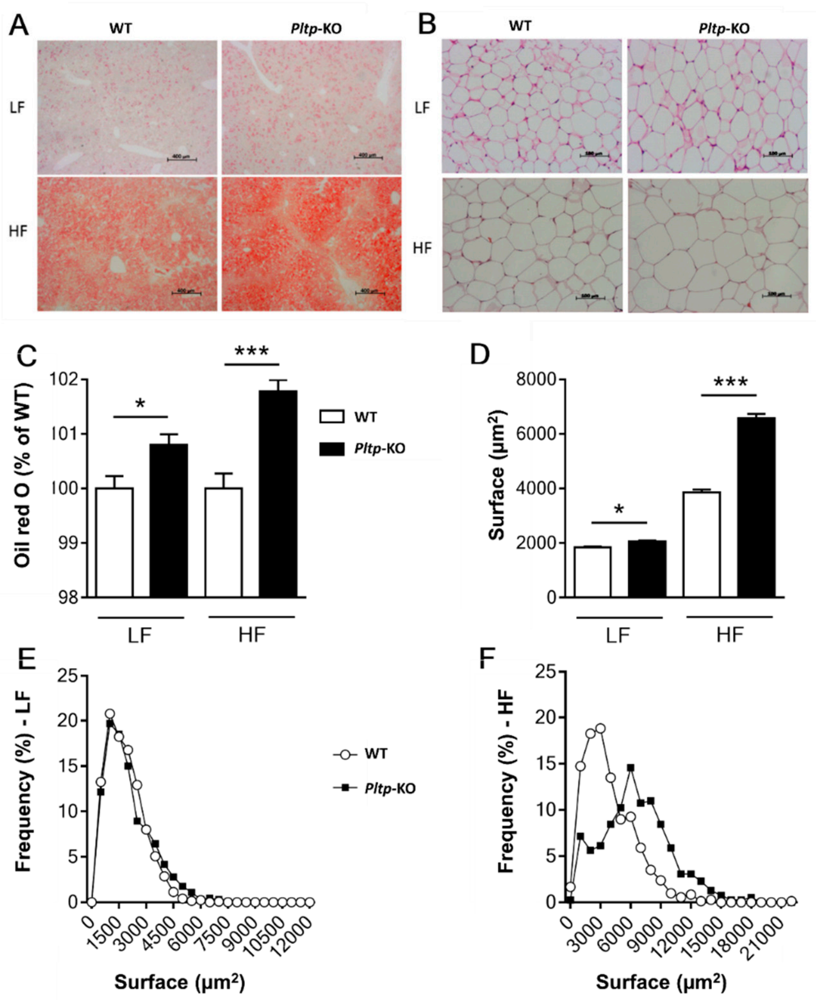

Figure S1. Tissue analyses (liver and adipose tissue) in WT and *Pltp*-KO mice under LF or HF. (**A**) Microscopy of mice liver (**B**). Microscopy of mice adipose tissue (**C**). Oil red O (% of WT) analyses in mice (**D**). Adipocyte surface in mice (µm²). (**E**). Distribution of adipocyte according to their surfaces in mice under LF. (**F**) Distribution of adipocytes according to their surfaces in mice under HF. Statistical analyses were performed using the Student Test, * $p < 0.05$ and *** $p < 0.001$. All results are expressed as mean ± SEM.
